# Identification of a Novel L-rhamnose Uptake Transporter in the Filamentous Fungus *Aspergillus niger*

**DOI:** 10.1371/journal.pgen.1006468

**Published:** 2016-12-16

**Authors:** Jasper Sloothaak, Dorett I. Odoni, Vitor A. P. Martins dos Santos, Peter J. Schaap, Juan Antonio Tamayo-Ramos

**Affiliations:** 1 Laboratory of Systems and Synthetic Biology, Wageningen University and Research, Stippeneng 4, Wageningen, The Netherlands; 2 LifeGlimmer GmbH, Markelstr. 38, 12163, Berlin, Germany; Oregon State University, UNITED STATES

## Abstract

The study of plant biomass utilization by fungi is a research field of great interest due to its many implications in ecology, agriculture and biotechnology. Most of the efforts done to increase the understanding of the use of plant cell walls by fungi have been focused on the degradation of cellulose and hemicellulose, and transport and metabolism of their constituent monosaccharides. Pectin is another important constituent of plant cell walls, but has received less attention. In relation to the uptake of pectic building blocks, fungal transporters for the uptake of galacturonic acid recently have been reported in *Aspergillus niger* and *Neurospora crassa*. However, not a single L-rhamnose (6-deoxy-L-mannose) transporter has been identified yet in fungi or in other eukaryotic organisms. L-rhamnose is a deoxy-sugar present in plant cell wall pectic polysaccharides (mainly rhamnogalacturonan I and rhamnogalacturonan II), but is also found in diverse plant secondary metabolites (e.g. anthocyanins, flavonoids and triterpenoids), in the green seaweed sulfated polysaccharide ulvan, and in glycan structures from viruses and bacteria. Here, a comparative plasmalemma proteomic analysis was used to identify candidate L-rhamnose transporters in *A*. *niger*. Further analysis was focused on protein ID 1119135 (RhtA) (JGI *A*. *niger* ATCC 1015 genome database). RhtA was classified as a Family 7 Fucose: H+ Symporter (FHS) within the Major Facilitator Superfamily. Family 7 currently includes exclusively bacterial transporters able to use different sugars. Strong indications for its role in L-rhamnose transport were obtained by functional complementation of the *Saccharomyces cerevisiae* EBY.VW.4000 strain in growth studies with a range of potential substrates. Biochemical analysis using L-[^3^H(G)]-rhamnose confirmed that RhtA is a L-rhamnose transporter. The RhtA gene is located in tandem with a hypothetical alpha-L-rhamnosidase gene (*rhaB*). Transcriptional analysis of *rhtA* and *rhaB* confirmed that both genes have a coordinated expression, being strongly and specifically induced by L-rhamnose, and controlled by RhaR, a transcriptional regulator involved in the release and catabolism of the methyl-pentose. RhtA is the first eukaryotic L-rhamnose transporter identified and functionally validated to date.

## Author Summary

The growth of filamentous fungi on plant biomass, which occurs through the utilization of its components (e.g. D-glucose, D-xylose, L-arabinose, L-rhamnose) as carbon sources, is a highly regulated event. L-rhamnose (6-deoxy-L-mannose) is a deoxy-sugar present in plant cell wall pectic polysaccharides (mainly rhamnogalacturonan I and rhamnogalacturonan II), but also in diverse plant secondary metabolites, ulvan from green seaweeds and glycan structures from virus and bacteria. The utilization, transformation or detoxification of this monosaccharide by fungi involves a first step of chemical hydrolysis, performed by alpha-L-rhamnosidases, and a second step of transport into the cell, prior to its metabolization. While many rhamnosidases have been identified, not a single eukaryotic plasma membrane L-rhamnose transporter is known to date. In this study we identified and characterized, for the first time, a fungal L-rhamnose transporter (RhtA), from the industrial workhorse *Aspergillus niger*. We also found that RhtA putative orthologs are conserved throughout different fungal orders, opening the possibility of identifying new transporters of its kind.

## Introduction

Organic carbon utilization by fungi is a biological process of great interest with many implications in ecology, agriculture and biotechnology. Their ubiquity and their ability to mobilize and metabolize a large variety of nutrients make fungi crucial players in the biogeochemical cycling of carbon in nature, in mutualistic symbiotic relationships with plants, and in pathogenic processes [1]. Their physiological resources for carbon utilization and biotransformation have also enhanced their relevance in the fields of (food) fermentation, bioindustrial chemistry and pharmacy, as they can be exploited for the production of enzymes, chemicals and other components of interest [2]. Thus, important efforts for the understanding of carbon utilization by fungi have been done, historically on those organisms that are genetically amenable, or have a direct impact on human affairs. In this sense, filamentous fungi from the *Aspergillus* genera, which include model species, species relevant for industrial applications, and human, animal and plant pathogens, have been deeply studied. In particular, the fungus *Aspergillus niger* has a versatile system for the degradation of the major plant cell wall polysaccharides: cellulose, hemicellulose and pectin, and due to its high enzyme secretory capacity is one of the main industrial producers of commercial enzymes for plant biomass conversion [3,4]. *A*. *niger* is able to synthetize an abundance of extracellular enzymes for lignocellulose depolymerization, and the encoding genes as well as the regulatory circuits that control their expression have been identified and characterized in detail [5–9]. Pectin utilization by fungi has received less attention, probably due to its structural complexity and to the nature of its polysaccharides. Pectin is composed of mainly D-galacturonic acid (approx. 65%), L-rhamnose, and branched with heterogeneous oligosaccharides. [10]. L-rhamnose is specifically found to be enriched in the pectic polysaccharide fractions rhamnogalacturonan I and II.

The pectin depolymerization enzyme network has been partly identified and characterized in several fungi [11–13], the catabolic pathways for the conversion of D-galacturonic acid and L-rhamnose have been genetically and biochemically characterized [14–17], and recently the transcription factors responsible for L-rhamnose and D-galacturonic acid utilization, RhaR and GaaR, have been identified [18,19]. Regarding the uptake of the specific pectin components, transporters responsible for the uptake of D-galacturonic acid have been reported in *A*. *niger* and *Neurospora crassa* [20,21]. However, not a single L-rhamnose transporter has been identified in fungi, or in any other eukaryotic organism. Besides its structural role in pectin, L-rhamnose is part of plant glycoproteins and secondary metabolites too, it is an important component of the green seaweed sulfated polysaccharide ulvan, and it is also present in glycan structures from virus and bacteria [22–24]. In prokaryotes, L-rhamnose uptake occurs via transporters from the RhaT family (2.A.7.6), which belongs to the drug/metabolite transporter superfamily (2.A.7) [25], but eukaryotic transporters belonging to the RhaT family have not been reported. Recently, an intracellular UDP-rhamnose/UDP-galactose transporter (nucleotide sugar transporter; NST), also member of the DMT superfamily, and located on the Golgi apparatus of *Arabidopsis thaliana*, was identified [26]. However NSTs are involved in transport processes related to the biosynthesis of plant cell wall components, and glycan structures, not found in eukaryotic plasma membranes, and not related to the uptake of sugars present in the environment. Monosaccharide transport by fungi has been investigated mainly in *Saccharomyces cerevisiae* in which transport of simple sugars is mediated only through facilitated diffusion by transporters from the sugar porter family, the largest within the major facilitator superfamily (MFS) [27]. The use of yeast monosaccharide transporter null mutants allowed for individual characterization of the individual transporters responsible for the uptake of D-glucose, D-fructose, D-mannose and D-galactose in yeast [27–29]. Yeast transporter null mutants were subsequently also used for the functional characterization of sugar porters from other fungal species [30–35].

The analyses of the global transcriptomic and proteomic responses of fungi to a variety of specific culture conditions are useful approaches to get insights in the specific structural and regulatory elements required for the utilization of specific carbon sources. In order to identify L-rhamnose transporter candidates, in this study a comparative plasmalemma proteome analysis was performed. The identification and functional validation of a eukaryotic L-rhamnose transporter is reported for the first time.

## Results

### Comparative plasmalemma proteome analysis for the identification of *A*. *niger* L-rhamnose transporter candidates

A recently developed approach for the study of the *A*. *niger* transportome through its plasma membrane proteomic analysis was used in order to perform a differential protein expression analysis of *A*. *niger* major facilitator superfamily (MFS) transporters. The approach is based on a LC-MS/MS analysis of plasmalemma enriched cellular fractions, and the method was applied to study the *A*. *niger* response to different D-glucose concentrations [36]. In the present study, a similar experimental set-up was used, but here the *A*. *niger* response to the presence of D-mannose, L-arabinose, D-xylose and L-rhamnose was studied. Mycelium of *A*. *niger* N400 was pre-grown for 18 h in minimal medium supplemented with 100 mM D-sorbitol as sole carbon source and equal amounts of biomass was transferred to controlled fermenters containing minimal medium with the following carbon source compositions: D-sorbitol 100 mM plus 0.1 mM D-xylose, 5 mM D-mannose, 5 mM L-arabinose or 5 mM L-rhamnose. The initial pH of these cultures was set at pH 4.0 and controlled at a lower limit of pH 3.5. Two hours after inoculation, mycelium was taken and cellular fractions enriched for plasma membranes were obtained as previously reported (see Materials and Methods and [36] for details). High-resolution analysis of the sugar content in the culture medium at the time of sampling showed that in all four conditions the respective sugars were being consumed (S1 Fig), so the presence of active transporters for the different monosaccharides could be expected.

For each culture condition peptide MS/MS spectra, obtained from the LC-MS/MS analysis of the enriched plasmalemma fractions, were processed as described in the Materials and Methods section. In total, 958 proteins were identified, of which 510 were present in all four conditions, while 41, 40, 65, and 43 proteins were present exclusively in the L-arabinose, D-mannose, L-rhamnose, and D-xylose conditions, respectively (S1 Dataset). The aim of this study was to identify specific L-rhamnose transporters, so the comparative proteome analysis was focussed on the identification and abundance analysis of candidate sugar porters. A total of 15 MFS transporters were identified in the presence of D-xylose, 16 in the presence of D-mannose, 19 in the presence of L-arabinose and 21 in the presence of L-rhamnose, of which 8 were shown to be exclusively detected in the L-rhamnose condition (Table 1). From these eight, a subgroup of five having strain ATCC 1015 [3] protein ID 1096151, 1119135, 1142034, 1147409 and 1156895 respectively were also absent in a previously generated dataset, where the *A*. *niger* plasmalemma proteome response to high and low concentrations of D-glucose was studied [36]. In summary, by analysing the *A*. *niger* plasma membrane proteome response to seven different carbon source compositions (D-sorbitol 100 mM, D-sorbitol 100 mM plus D-glucose 1 mM, D-sorbitol 100 mM plus D-glucose 60 mM, D-sorbitol 100 mM plus D-xylose 0.1 mM, L-arabinose 5 mM, D-mannose 5 mM, and L-rhamnose 5 mM) five putative transport proteins could be identified that were present only in the presence of L-rhamnose, which strongly suggested involvement of one or more of these transporters in the uptake of L-rhamnose.

**Table 1:** Relative abundance of MFS transporter proteins detected in different *A*. *niger* growth conditions

Prot ID	Relative abundance ± sd (%) x 100			
	L-arabinose	D-mannose	L-rhamnose	D-sorbitol + D-xylose
1089440	n.d.	n.d.	1.91 ± 0.98	n.d.
**1096151**	n.d.	n.d.	4.20 ± 2.40	n.d.
1101809	5.12 ± 1.73	1.60 ± 0.08	7.94 ± 0.90	2.95 ± 0.24
1105147	6.20 ± 0.21	8.13 ± 0.82	11.78 ± 0.22	6.78 ± 0.44
1105500	0.54 ± 0.20	n.d.	n.d.	n.d.
1111630	0.49 ± 0.10	1.71 ± 0.15	1.31 ± 0.14	1.38 ± 0.45
**1119135;** **(RhtA)**	n.d.	n.d.	10.94 ± 4.21	n.d.
1121621	3.64 ± 1.31	5.90 ± 0.73	n.d.	4.96 ± 0.48
1122202	0.63 ± 0.20	0.92 ± 0.05	0.46 ± 0.19	0.28 ± 0.01
1125086	37.93 ± 5.96	n.d.	n.d.	n.d.
1128338	n.d.	4.98 ± 1.11	n.d.	2.03 ± 0.07
1129336	n.d.	0.39 ± 0.08	n.d.	0.26 ± 0.01
1142034	n.d.	n.d.	2.26 ± 0.04	n.d.
1142882	2.95 ± 0.32	2.30 ± 0.56	4.40 ± 0.11	2.21 ± 0.50
**1143191**	n.d.	n.d.	7.04 ± 0.97	n.d.
1143598	3.34 ± 0.89	4.59 ± 2.03	6.51 ± 5.28	8.51 ± 0.12
1144375	5.80 ± 2.89	0.72 ± 0.23	15.25 ± 3.84	n.d.
1144791	16.06 ± 4.06	n.d.	17.75 ± 2.24	6.20 ± 1.05
**1147409**	n.d.	n.d.	0.78 ± 0.44	n.d.
**1156895**	n.d.	n.d.	10.92 ± 0.57	n.d.
1160647	2.47 ± 1.61	n.d.	n.d.	n.d.
1164538; 1188786*	0.55 ± 0.11	1.30 ± 0.12	1.03 ± 0.24	0.9 5± 0.01
1165706	n.d.	n.d.	n.d.	0.06 ± 0.05
1167504	1.07 ± 0.09	0.39 ± 0.12	n.d.	n.d.
1169204	0.54 ± 0.13	n.d.	n.d.	5.07 ± 0.95
1178623	7.53 ± 2.54	5.20 ± 0.00	5.64 ± 4.12	n.d.
1180703	n.d.	n.d.	2.61 ± 0.25	n.d.
1188093	0.85 ± 0.08	0.96 ± 0.15	1.59 ± 0.15	0.78 ± 0.16
1188840	n.d.	0.60 ± 0.24	n.d.	n.d.
1189214	35.93 ± 0.54	n.d.	2.87 ± 1.02	n.d.

n.d.: not detected

*: same protein group; ProtID underlined indicate putative transporters detected in L-rhamnose, but not in L-arabinose, D-mannose and D-xylose conditions. ProtID in bold indicate putative transporter proteins not detected as well in the previous proteomic analysis performed by Sloothaak et al. (2015), where *A*. *niger* was grown in the presence of D-sorbitol 100 mM, D-sorbitol 100 mM plus D-glucose 1 mM, and D-sorbitol 100 mM plus D-glucose 60 mM [36]. ProtIDs were obtained from the JGI ATCC1015 database [37].

A detailed protein sequence analysis of these transporters and of their encoding genes highlighted some interesting features of protein ID 1119135, hereinafter referred to as RhtA for Rhamnose transporter A. Domain analysis of the transporters revealed that RhtA was exceptional because it possessed a L-fucose permease domain structure (FucP; COG0738/IPR005275). FucP domain transporters have been shown to be able to use different deoxy sugars as substrate, such as L-fucose, 2-deoxy-D-ribose and 2-deoxy-D-glucose, but also monosaccharides such as L-arabinose, D-galactose, D-glucose and D-mannose [38–40]. To our knowledge transporters with a FucP domain structure have only been characterized from bacteria. However, the particular domain structure is well represented throughout the fungal kingdom. Through a Bi-directional Best Hit (BBH) BLAST analysis, putative RhtA orthologs were inferred. Homologous sequences were found throughout different fungal orders, but only 5 BBH’s were identified when analyzing the available genomes of *Aspergillus* spp. (S1 Table).

**Fig 1:**
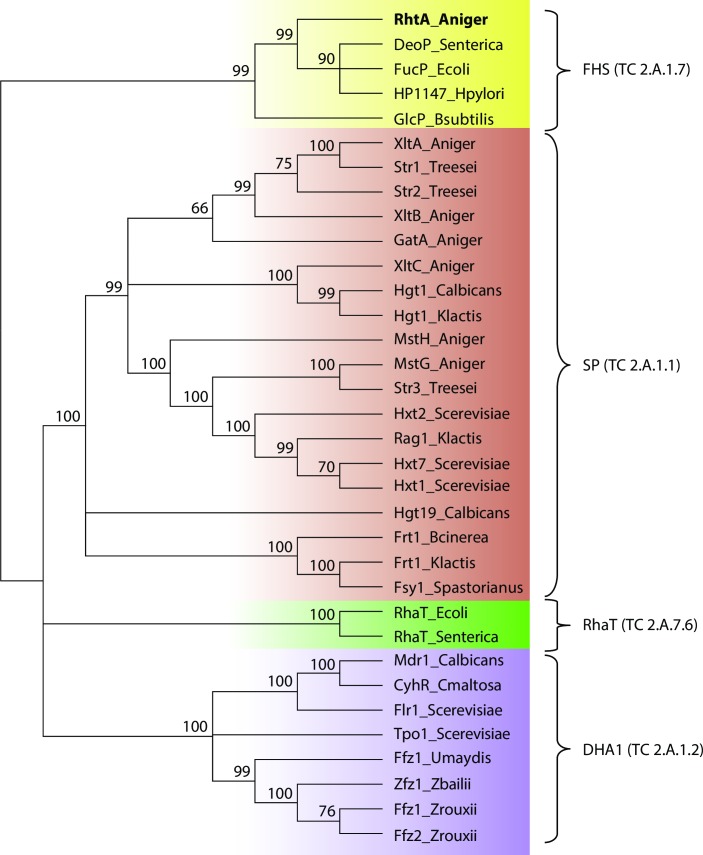
Classification of *A*. *niger* RhtA. Sequences of biochemically characterized sugar transporters were collected and a multiple sequence alignment was created using Praline alignment suite, which takes secondary structure predictions into account [83]. A neighbour-joining tree was then generated with 1000 bootstrap replicates.

A domain based classification of RhtA, using amino acid sequences of 27 functionally validated fungal MFS sugar transporters, 2 bacterial L-rhamnose transporters, and 4 characterized bacterial FucP domain symporters as input, grouped the RhtA transporter with the latter group (Fig 1). These transporters belong to the Fucose:H1 symporter (FHS) family (TC 2.A.1.7) within the Major Facilitator Superfamily [41] (Fig 1). The domain architecture COG0738 is defined by 13 reference sequences, of which *Bacillus subtilis* GlcP, *Escherichia coli* FucP and *Helicobacter*. *pylori* HP1174 [38,39,42] have been characterized, while DeoP from *Salmonella enterica* is probably a 2-deoxy-D-ribose permease [40]. *E*. *coli* FucP transports L-fucose, L-arabinose and D-galactose but it is not able to transport L-rhamnose, *B*. *subtilis* GlcP has high affinity for D-glucose and D-mannose, and *H*. *pylori* HP1174 is able to use D-glucose, D-galactose, D-mannose and 2-deoxy-D-glucose as substrates. L-fucose and L-rhamnose are structurally related, as both of them are methyl pentoses, however there have been no reports on the ability of FHS symporters to use L-rhamnose as substrate, for which specific transporters from the L-rhamnose proton symport family (RhaT; cl05728/IPR004673) have been described in bacteria [43]. Characterized bacterial L-rhamnose transporters from the L-rhamnose transporter family (RhaT) (TC 2.A.7.6) clustered together in a separate subgroup in the phylogenetic tree (Fig 1). This was expected because they are not related to the MFS (2.A.1) and have no similarities with proteins from this superfamily [44]. The remaining fungal functionally validated sugar transporters are clustered in two additional subgroups corresponding to other MFS families: the sugar porter (SP) family (TC 2.A.1.1) and the drug:H1 antiporter-1 (12 spanner) (DHA1) (TC 2.A.1.2) (Fig 1).

Analysis of the genomic *locus* of the RhtA encoding gene revealed directly upstream of *rhtA* an ORF coding for a hypothetical secreted glycoside hydrolase, hereinafter referred to *rhaB*, from the GH78 family (Protein ID 131668) which only includes α-L-rhamnosidases. The hypothetical RhaB is a 832 amino acid protein with low similarity to functionally validated α-L-rhamnosidases. However, its amino acid number is in the range of RhaE from *A*. *nidulans* (861 aa), and many of the characterized bacterial rhamnosidases [13]. The *rhaB-rhtA* tandem localization, which could conform a L-rhamnose uptake system in *A*. *niger*, was found to be present as well in *Aspergillus luchuensis* and *Aspergillus kawachii*, which have been reported to be the same species [45], and are closely related to *A*. *niger*.

Taking the above-described findings into account, RhtA was considered a strong candidate to be a transporter specific for L-rhamnose. In order to validate this hypothesis, and to unravel the function of this eukaryotic transporter with a FucP domain signature, RhtA was selected for functional validation and characterisation in the present study.

### Functional validation of the *A*. *niger* sugar transporter RhtA

In order to test the functionality of the RhtA transporter, the engineered *S*. *cerevisiae* strain EBY.VW4000, a monosaccharide transporter null mutant, was chosen as host for the heterologous expression of the *rhtA* coding gene. *S*. *cerevisiae* is not able to use L-rhamnose as a carbon source, so a direct functional complementation approach based on the use of this deoxy sugar could not be performed with this strain. Despite this, and as discussed below, heterologous expression of the transporter in this genetic background gave clear insights about the possible role of RhtA on L-rhamnose transport.

**Fig 2:**
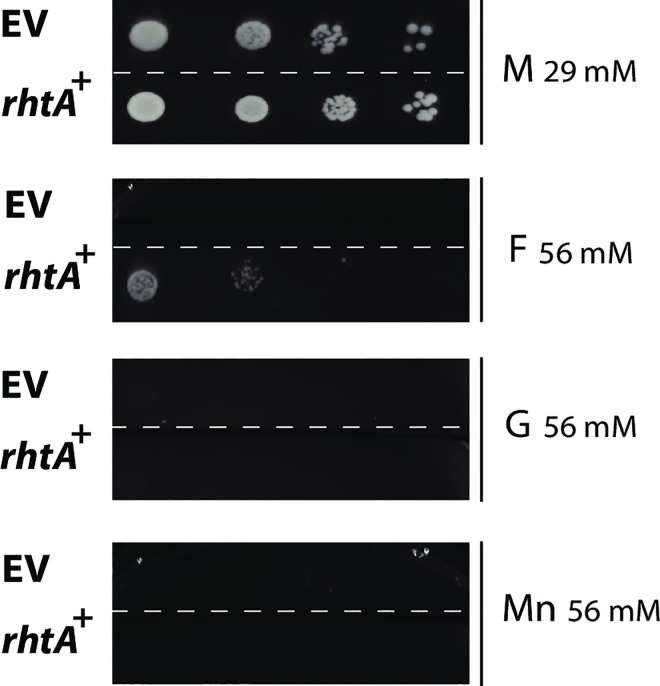
RhtA functional analysis in yeast. Growth of strain EBY.VW4000 expressing the *rhtA* gene (*rhtA*^+^) or harbouring the empty expression vector p426HXT7-6His (EV) in minimal medium agar plates containing maltose (M; 29 mM), D-glucose (G; 56 mM), D-fructose (F; 56 mM) or D-mannose (Mn; 56 mM) as sole carbon sources. Agar plates were incubated at 30°C for 96 h. Transformants expressing RhtA showed the same growth pattern; the figure depicts only one representative transformant.

**Fig 3:**
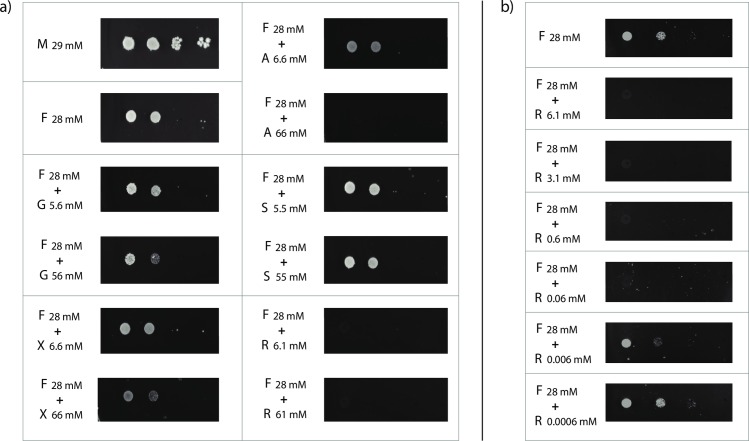
EBY.VW4000 *rhaA*^+^ growth inhibition assays. a) Growth of yeast strain EBY.VW4000 expressing the *rhtA* gene on minimal medium with maltose (M; 29 mM), D-fructose (F; 28 mM), and D-fructose (F; 28 mM) supplemented with the potentially competing carbon sources: D-glucose (G; 56 mM and 5.6 mM), D-xylose (X; 66 mM and 6.6 mM), L-arabinose (A; 66 mM and 6.6 mM), D-sorbitol (S; 55 mM and 5.5 mM) or L-rhamnose (R; 61 mM and 6.1 mM)**; b)** growth of yeast strain EBY.VW4000 expressing the *rhtA* gene on minimal medium with D-fructose (F; 28 mM), and D-fructose (F; 28 mM) supplemented with a range of L-rhamnose concentrations (0.0006 mM to 6.1 mM).

**Fig 4:**
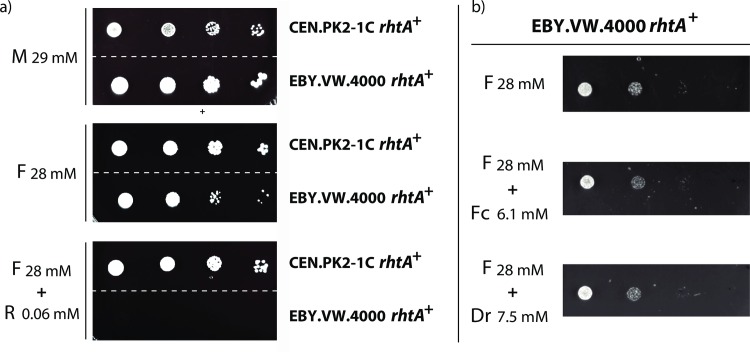
EBY.VW4000 *rhaA*^+^ and CEN.PK2-1C *rhaA*^+^ growth inhibition assays. a) Growth of yeast strains CEN.PK2-1C and EBY.VW4000 expressing the *rhtA* gene on minimal medium with maltose (M; 29 mM), D-fructose (F; 28 mM) or D-fructose (F; 28 mM) supplemented with L-rhamnose (R; 0.06 mM); b) Growth of EBY.VW4000 *rhaA*^+^ on minimal medium containing D-fructose (F; 28 mM) or D-fructose (F; 28 mM) supplemented with L-fucose (Fc; 6.1 mM) or 2-deoxy-D-ribose (Dr; 6.1 mM).

The yeast strain was transformed with the 2μ expression plasmid p426HXT7-6His-rhtA, containing the gene’s cDNA under control of the constitutive promoter HXT7_p_ and the terminator CYC1_t_. Single colony transformants were isolated from minimal medium agar plates containing 2% (w/v) maltose and the ability of *rhtA* to restore growth of the EBY.VW4000 transformant strain in the presence of different monosaccharides was studied. Ten-fold serial dilutions of logarithmically growing cells from at least three different transformants expressing *rhtA* were spotted on different minimal medium plates supplemented with 1% (w/v) of the following carbon sources: D-glucose (G; 56 mM), D-fructose (F; 56 mM), D-mannose (Mn; 56 mM) and maltose (M; 29 mM). Yeast *rhtA* transformants showed an ability to restore growth on D-fructose, albeit at a slow pace, but were not able to restore growth on D-glucose and D-mannose (Fig 2). This result indicated that RhtA was functional as a transporter in *S*. *cerevisiae*, but as expected none of the substrates tested seemed to be ideal for this transporter. The fact that D-fructose was used as a substrate by RhtA allowed us to perform sugar competition assays on plate, which gave more insights in possible additional substrates for this transporter. Hence, the ability of an *rhtA* transformant to grow in the presence of D-fructose (F; 28 mM) was compared to its ability to grow in plates containing D-fructose (F; 28 mM) mixed with either D-glucose (G; 56 mM or 5.6 mM), D-xylose (X; 66 mM or 6.6 mM), L-arabinose (A; 66 mM or 6.6 mM), D-sorbitol (S; 55 mM or 5.5 mM) or L-rhamnose (R; 61 mM or 6.1 mM). The *rhtA* transformant strain was able to grow in the presence of most of the sugar mixes tested, but it was unable to grow on D-fructose in the presence of a high and low concentration of L-rhamnose, suggesting that D-fructose uptake by RhtA was strongly inhibited by L-rhamnose (Fig 3A). D-fructose uptake by RhtA was also inhibited by L-arabinose, at a concentration of 66 mM, but not at a concentration of 6.6 mM. The results suggested that RhtA could have a higher affinity for L-rhamnose than for any of the other sugars tested. To determine the lower boundary for D-fructose uptake inhibition, the experiment was then repeated with lower L-rhamnose concentrations (0.0006 mM to 6.1 mM) in the presence of D-fructose (28 mM) as carbon source (Fig 3B). As shown in Fig 3B, growth was inhibited by L-rhamnose concentrations as low as 0.06 mM. The fact that such a low concentration of L-rhamnose, around 500 times lower than of D-fructose, was able to inhibit growth, pointed to the deoxy sugar as a possible true substrate of the RhtA transporter. To prove that the strong growth inhibitory capacity of L-rhamnose was exclusively associated to the RhtA transporter, and not due to an unexpected toxicity of L-rhamnose on the yeast itself, a different strain (CEN.PK2-1C), isogenic to EBY.VW4000, harboring the p426HXT7-6His-rhtA, was also grown in the same conditions. The CEN.PK2-1C strain expressing RhtA was able to grow in the presence of D-fructose 28 mM + L-rhamnose 0.06 mM (Fig 4A), thus indicating that the D-fructose uptake inhibition invoked by L-rhamnose was RhtA dependent, as inhibition of growth did not occur in the yeast strain with functional endogenous D-fructose transport systems.

Finally, a competition plate assay was performed using two alternative deoxy sugars: L-fucose (Fc; 6.1 mM) and 2-deoxy-D-ribose (Dr; 7.5 mM). As it can be observed in (Fig 4B) the RhtA transformant showed normal growth. Taken together, these results suggested that *A*. *niger* RhtA could be a very specific transporter for L-rhamnose, unable to use other deoxy sugars as substrates.

### Transcriptional analysis of the *A*. *niger* sugar transporter coding gene *rhtA*

**Fig 5:**
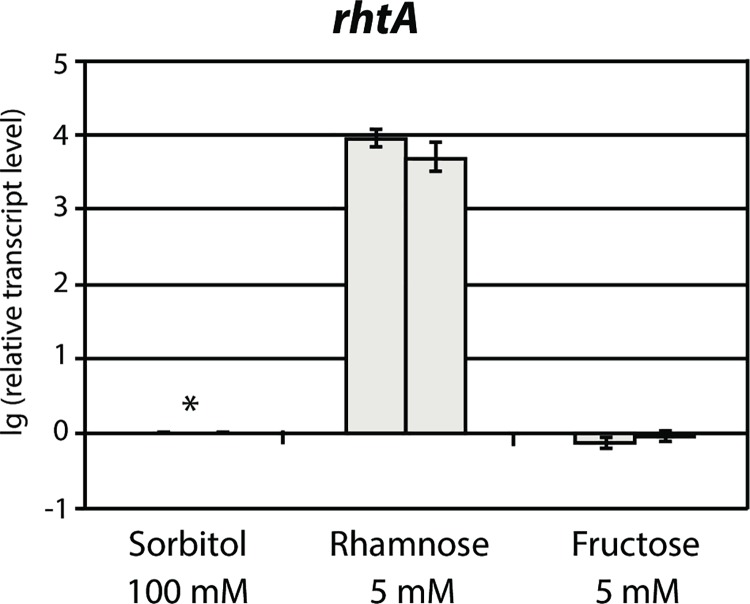
Transcriptional analysis of *rhtA*. Relative transcription levels were measured by RT-qPCR, in *A*. *niger* N400 sampled 2 hours after mycelium transfer to minimal medium with 100 mM D-sorbitol (reference), 5 mM L-rhamnose or 5 mM D-fructose. Transcript levels are relative to reference sample (D-sorbitol 100 mM), indicated with an asterisk. Results are given as relative transcript ratios in logarithmic scale (lg(10)). The values provided in the figures correspond to two biological replicates per culture condition. Error bars are means of three technical replicates.

According to the RhtA protein abundance profile obtained by plasmalemma proteome analysis, the expression of the transporter seemed to be specifically induced by L-rhamnose. This fact, together with the additional findings described above, indicated that RhtA could be a L-rhamnose transporter. However, in yeast complementation experiments RhtA showed an ability to transport D-fructose, so in *A*. *niger* the transporter’s biological role could be related to D-fructose uptake as well. Having this into account, new fermentations of the *A*. *niger* N400 wild type strain were performed, using the same set-up as described above, in order to study the transcriptional response of *rhtA* to D-sorbitol 100 mM (reference), L-rhamnose 5 mM, and D-fructose 5 mM. Samples, obtained two hours after mycelium transfer, were processed, and RT-qPCR analysis was performed. As expected, the *rhtA* gene was strongly induced in the presence of L-rhamnose, while its expression levels in the presence of D-fructose were found to be similar to those observed in the reference condition (Fig 5). This result showed that L-rhamnose, but not D-fructose, acts as an inducer of the RhtA transporter at transcriptional level, suggesting that RhtA is not a natural D-fructose transporter.

**Fig 6:**
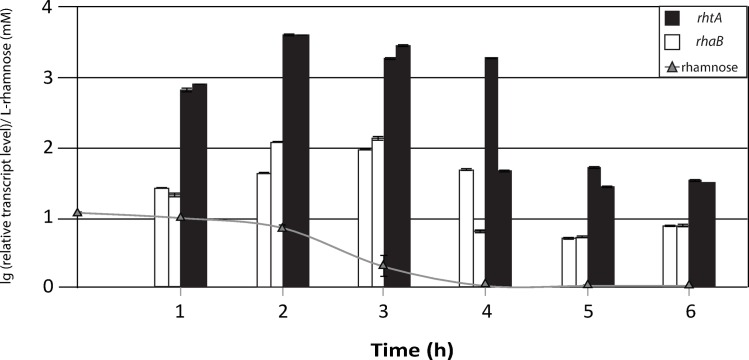
Time course transcriptional analysis of *rhtA* and *rhaB*. Relative transcription levels, measured by RT-qPCR, of *rhtA* (black bars) and *rhaB* (white bars) during *A*. *niger* N400 fermentations in minimal medium with an initial concentration of L-rhamnose 1 mM. Concentration of L-rhamnose over time is represented by grey line with triangles (concentration at t = 4h is equal to 0). Transcript levels of both genes always refer to the reference sample (D-sorbitol 100 mM; t = 1h). Results are given as relative transcript ratios in logarithmic scale (lg(10)). The values provided in the figures correspond to two biological replicates per culture condition. Error bars are means of three technical replicates.

As mentioned above, the gene *rhaB*, coding for a hypothetical α-L-rhamnosidase (JGI *A*. *niger* ATCC 1015 Protein ID 131668), was found to be located directly upstream of *rhtA*. The amino acid analysis of the hypothetical α-L-rhamnosidase revealed the presence of a classical N-terminal secretion signal peptide. Thus, it would be possible that both *rhaB* and *rhtA* gene products have a coordinated action, releasing and transporting L-rhamnose monomers respectively. This would imply that both proteins are coordinately expressed in the presence of the sugar. To obtain more insight into the transcriptional responses of *rhtA* and *rhaB* to L-rhamnose, time course fermentations were done, in cultures containing either D-sorbitol 100 mM, L-rhamnose 1 mM or L-rhamnose 5 mM as sole carbon source. Sampling was performed every hour after mycelium transfer, until the L-rhamnose was depleted from the medium for two hours. The RT-qPCR results obtained showed a fast transcriptional response of *rhtA* to the presence of L-rhamnose (Fig 6), while in the D-sorbitol reference condition *rhtA* expression remained at low level (S2 Fig). One hour after mycelium transfer the *rhtA* expression level in the presence of an initial L-rhamnose concentration of 1mM was approximately 1000 fold higher than those observed in the reference condition (D-sorbitol 100 mM; t = 1h). A maximum was observed two hours after transfer, with *rhtA* transcriptional levels being 5000 fold higher than in the reference condition. The *rhtA* transcription profile for the first three hours was the same in the 5 mM L-rhamnose fermentation (S2 Fig). The maximum *rhtA* expression levels remained constant during the time course experiment until the deoxy sugar was completely consumed (Fig 6). After this point, *rhtA* mRNA levels decreased approximately 80 fold. Once the *rhtA* mRNA levels decreased, they remained constant at least 2 hours, being still around 50 times higher than its expression levels in the reference condition. High mRNA stability could be the reason why the *rhtA* expression levels kept being relatively high, even several hours after the sugar was completely consumed.

The results displayed in Fig 6 show that *rhaB* has a similar expression profile to the one observed for *rhtA*: its expression was activated in the presence of L-rhamnose and remained constant until the sugar was depleted from the medium (reference condition: D-sorbitol 100 mM; t = 1h). Therefore both genes seem to have a specific coordinated response to the presence of L-rhamnose in the environment.

**Fig 7:**
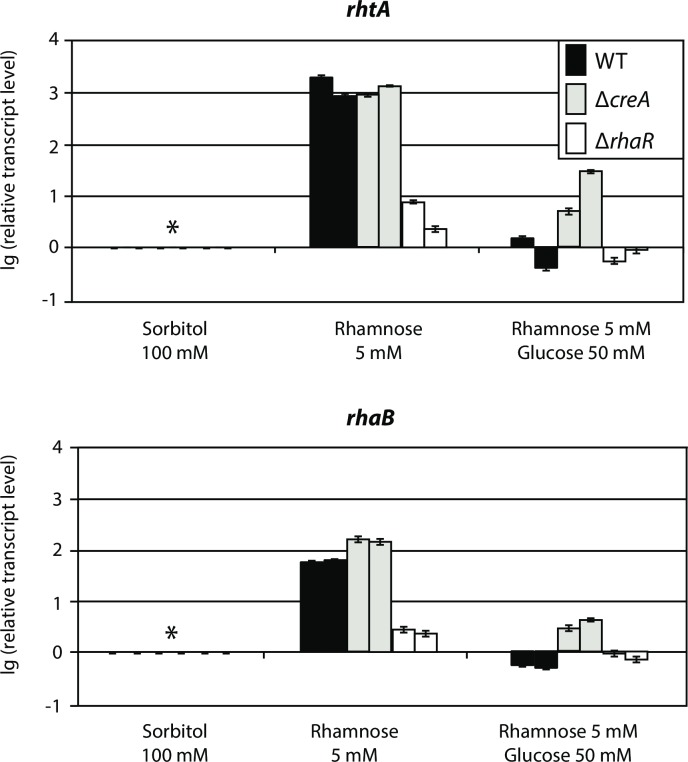
Role of the CreA and RhaR transcriptional regulators on the expression of *rhtA* and *rhaB*. Strains N402 (WT; black bars), NW283 (*ΔcreA;* grey bars), and JS014 (*ΔrhaR*; open bars) were used. Relative transcription levels were measured by RT-qPCR in samples obtained 2 hours after mycelium transfer to 5 mM L-rhamnose or 5 mM L-rhamnose plus 50 mM D-glucose. Relative transcript levels of *rhtA* and *rhaB* were calculated using the pre-culture condition of each strain (D-sorbitol 100 mM; t = 18h), sampled prior to the mycelium transfer to inducing and inducing/repressing conditions, as reference (*). Results are given as relative transcript ratios in logarithmic scale (lg(10)). The values provided in the figures correspond to two biological replicates per culture condition. Error bars are means of three technical replicates.

To further understand the regulatory mechanisms underlying the L-rhamnose uptake system encoded by the *rhtA* and *rhaB* genes, the role of the regulators RhaR, involved in L-rhamnose release and catabolism [18], and CreA, mediating carbon catabolite repression in plant cell wall utilisation systems [46], was studied. To do this, a transcriptional analysis of both genes in the strains N402 (WT), NW283 (*ΔcreA*) and JS14 (*ΔrhaR*) (see Materials and Methods section for construction details) was done. The wild type and the *ΔcreA* and *ΔrhaR* strains were pre-cultured in MM with 100 mM D-sorbitol for 18 h, and transferred to MM with either 5 mM L-rhamnose or 5 mM L-rhamnose plus 50 mM D-glucose. Samples were taken two hours after mycelium transfer, and were subsequently processed for RT-qPCR analysis. As previously observed, both *rhtA* and *rhaB* were induced by L-rhamnose. Their transcriptional levels in the presence of the deoxy sugar were similar in both the wild type (N402) and *ΔcreA* (NW283) strains (Fig 7). In the presence of L-rhamnose plus D-glucose, *rhtA* and *rhaB* were heavily repressed in the wild type strain, and only partly derepressed in the *ΔcreA* mutant. Regarding the regulatory mechanisms mediating the activation of *rhtA* and *rhaB*, the expression of both genes in the presence of L-rhamnose was strongly reduced in the *ΔrhaR* strain, suggesting that the RhaR transcriptional activator is responsible for the induction of these genes.

### Determination of the RhtA ability to transport L-rhamnose

**Fig 8:**
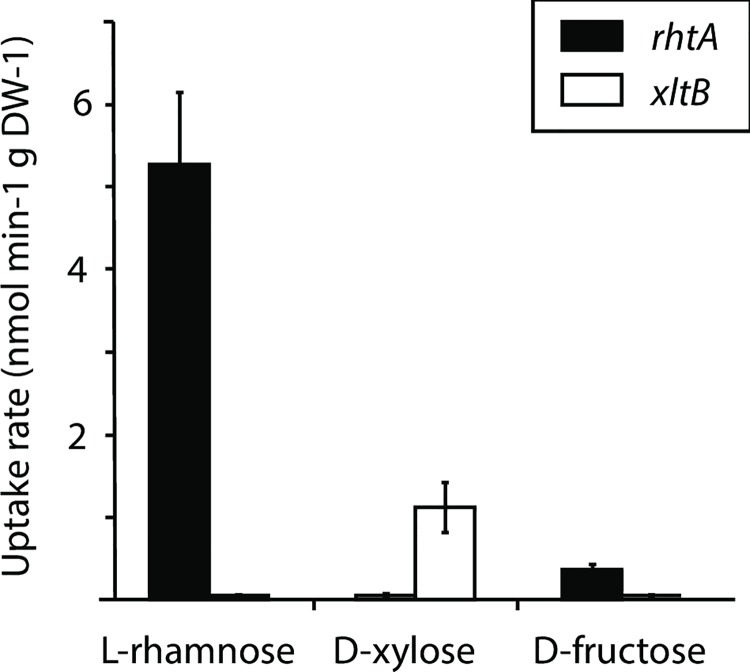
Functional characterization of *A*. *niger* RhtA sugar transporter. Uptake at high cell density of radiolabeled substrates by *S*. *cerevisiae* EBY.VW4000 expressing the *Aspergillus niger* L-rhamnose transporter gene *rhtA* (black bars) or D-xylose transporter gene *xltB* (open bars). Radiolabeled L-rhamnose, D-xylose or D-fructose were added at a final concentration of 20 μM.

To prove that RhtA can use L-rhamnose as substrate, a tritium labeled L-[^3^H(G)]-rhamnose uptake experiment was performed. In this experiment, the L-rhamnose uptake ability of the EBY.VW4000_RhtA strain was determined, using as a negative control the EBY.VW4000 strain expressing the *A*. *niger* specific D-xylose transporter XltB [47]. Additionally, to further investigate the RhtA transporter selectivity, D-[1-^14^C]-xylose and D-[^14^C(U)]-fructose were also tested as possible substrates. As shown in Fig 8, the RhtA strain showed a L-rhamnose uptake rate of 5.28×10^−3^ ± 0.87×10^−3^ nmol min^-1^ mg DW^-1^, while the transport of the methyl-pentose by the control strain could not be detected. This result, together with the previous findings reported in this study, confirmed that RhtA is a functional L-rhamnose transporter in *A*. *niger*. As expected, the control strain EBY.VW4000_XltB strain was able to transport D-xylose, with an uptake rate of 1.12×10^−3^ ± 0.31×10^−3^ nmol min^-1^ mg DW^-1^, while the RhtA expressing strain could not. Finally, radiolabeled D-fructose uptake measurements confirmed the observations previously done in the functional complementation studies performed for XltB and RhtA: the EBY.VW4000_RhtA transformant could transport D-fructose with an uptake rate of 0.37×10–3 ± 0.06×10^−3^ nmol min^-1^ mg DW, while D-fructose uptake by the XltB strain could not be detected. The L-rhamnose uptake rate determined for RhtA is significantly lower than that one reported for the *E*. *coli* L-rhamnose transporter [43], however, the experimental approach used by Tate et al., which performed overnight cultivations of different *E*. *coli* strains (WT and L-rhamnose negative strains) for that purpose, make a comparison difficult. The L-rhamnose uptake rate determined for RhtA was, however, comparable to the maximum sugar uptake rate determined for other fungal MFS transporters, like the D-xylose transporters GXS1 from *Candida intermedia* [48] or XylH from *Debaryomyces hansenii* [49].

### Deletion analysis of the *A*. *niger rhtA* and *rhaB* genes

**Fig 9:**
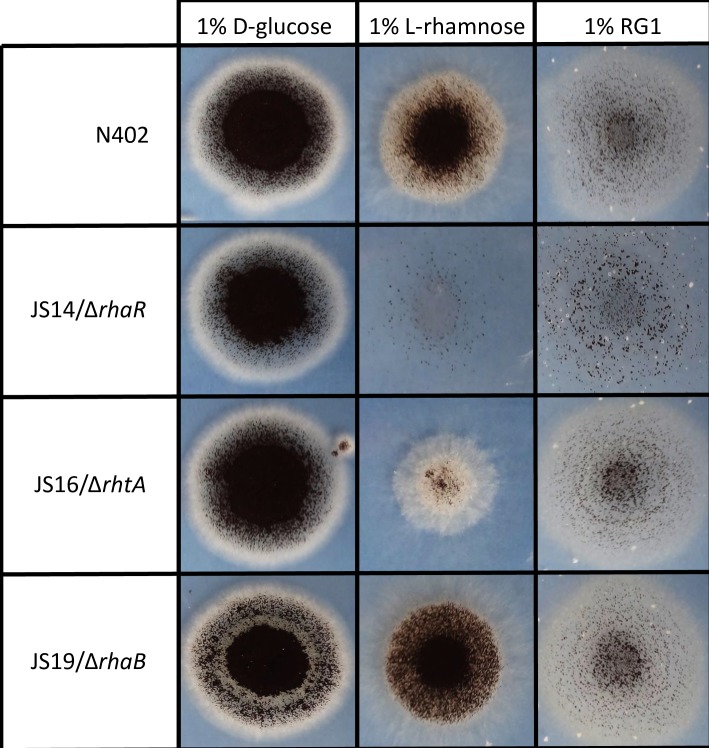
Phenotype analysis of *A*. *niger* strains N402 (WT), JS14 (Δ*rhaR*), JS16 (Δ*rhtA*) and JS19 (Δ*rhaB*). *A*. *niger* strains were plated on minimal media supplemented either with D-glucose (1%; w/v), L-rhamnose (1%; w/v) or rhamnogalacturonan I (RG1) (1%; w/v) as sole carbon source, and cultured for 144 hours. Mutants with the same gene deleted showed the same growth pattern; the figure depicts only one representative knockout strain per gene.

To further understand the biological role of the L-rhamnose transporter and the hypothetical α-L-rhamnosidase in *A*. *niger*, deletion strains of *rhtA* (JS16) and *rhaB* (JS19) were constructed (see Materials and Methods section for construction details). In each case, two knockout strains were isolated, and their growth phenotype was studied by plating them on minimal media supplemented either with D-glucose (1% w/v), L-rhamnose (1% w/v) or rhamnogalacturonan I (RG1) (1% w/v) as sole carbon source. The N402 wild type strain and the regulator mutant Δ*rhaR* were used as controls. Growth in D-glucose was comparable, whereas clear differences could be observed in L-rhamnose plates (Fig 9). In the presence of of the methyl-pentose, the Δ*rhaR* mutants did not grow (as was described by Gruben et al. (2014) [18]). The two Δ*rhtA* mutants were severely affected, showing less growth and sporulation, while the Δ*rhaB* transformants showed a normal growth. The growth reduction observed with the Δ*rhtA* mutants suggests a relevant role for RhtA in L-rhamnose uptake. The fact that the Δ*rhtA* mutants are still able to grow in the presence of L-rhamnose as sole carbon source indicates that *A*. *niger* must possess at least one other transporter capable of transporting the monosaccharide. All strains grew poorly in the presence of RG1, although slightly less growth could be observed in the Δ*rhaR* mutant.

## Discussion

The prediction of substrate specificity of (sugar) transporters by using general classification systems is difficult, and traditional methods based on shared primary sequence similarity (e.g. the standard Blast algorithm) are in many cases not precise enough [47,50]. The use of profile hidden Markov models (HMM) to segregate sugar transporter proteins based on their substrate has been shown as an effective approach, however their precision largely depends on the availability of a consistent training set of biochemically characterized proteins with the function of interest [36,47]. In this study we aimed, for the first time, to identify a eukaryotic L-rhamnose transporter and consequently, no eukaryotic examples of L-rhamnose transporters were available in the literature. Bacterial examples of L-rhamnose transporters from *E*. *coli* and *Salmonella typhimurium* identified in the early 1990s [43] belong to the TC 2.A.7.6 transporters family, which has no similarity with MFS transporters, and have not been associated to the transport of sugars in eukaryotic organisms. To identify putative transporters specific for L-rhamnose we have taken advantage of the fact that *A*. *niger* possesses complex regulatory circuits that control tightly the expression of protein sets, including extracellular enzymes, transporter proteins and metabolic enzymes, specific for the utilization of different sugars [7]. Therefore, the analyses of global transcriptomic and proteomic responses of the fungus to a variety of specific culture conditions can be useful approaches to get insights in the specific structural elements, including specific sugar transporters, required for the utilization of specific carbon sources. The activation of structural genes involved in L-rhamnose utilization by *A*. *niger* and other fungi have been shown to require the presence of an inducing carbon source which can be either L-rhamnose or pectic polysaccharides [16,18,51]. Assuming that the above also holds for L-rhamnose specific transporters, RhtA was selected by applying stringent differential protein expression criteria. Heterologous expression of candidate transporters in the *S*. *cerevisae* monosaccharide transporter null strain EBY.VW4000 has been a very effective tool to study the function of single monosaccharide transporters in isolation. Although this strain naturally only utilizes hexoses like D-glucose and D-fructose, it has been amended to metabolize D-xylose or L-arabinose [52–54]. Accordingly, the strain can be used to screen for L-arabinose and D-xylose transporters. In a similar manner, a microbial L-rhamnose utilisation pathway transferred to the EBY.VW4000 strain would allow screening for L-rhamnose transporters. By using an alternative approach, we managed to get important insights in the possible role of *A*. *niger* RhtA as a functional L-rhamnose transporter. First, we showed that RhtA is expressed as a functional transporter able to transport D-fructose. Subsequently, we determined that L-rhamnose had an extraordinary ability to specifically inhibit growth of the EBY.VW4000_RhtA strain, even at micromolar levels. This fact, plus additional insights observed at genomic, transcriptomic and proteomic level gave strong indications that RhtA acts as a specific transporter for L-rhamnose, but did not provide direct evidence. To assay transport capacity and quantify uptake kinetics, radiolabeled sugar uptake experiments can be performed with the mentioned yeast strain [33,34,36,55–57]. This approach was followed in the present study as a final step to prove the ability of RhtA to transport L-rhamnose.

Besides the ability of RhtA to transport L-rhamnose, we have obtained important insights into the biological role of the transporter. The tandem localization of *rhtA* with the α-L-rhamnosidase coding gene *rhaB*, and their coordinated transcriptional response to the presence of L-rhamnose, indicated that both proteins could have a joint action in releasing and transporting the sugar. We also investigated if, besides *rhaB*, additional hypothetical α-L-rhamnosidase genes (1180185/An08g09140, 1160525/An18g04800, 1132057/An01g06620, 1180185/An15g04530, 1126821/An04g09070, 1134376/An07g00240, and 1165677/An10g00290; Protein accession numbers in ATCC 1015 and CBS 513.88 genomes) were co-localized with sugar transporters and noticed that the co-expressed *rhaB*-*rhtA* tandem was a unique case in *A*. *niger*.

The transcriptional profile of *rhtA* and *rhaB* genes also suggests a coordinated role for the utilization of the deoxy sugar. In the presence of an initial L-rhamnose concentration of 1 mM their expression was strongly induced while the sugar was being consumed. Concentrations even lower than 1 mM might therefore also induce this system, as it occurs in the case of the D-xylose utilisation system in *A*. *niger*, where a concentration of 0.1 mM D-xylose already exerts a strong activation of structural genes like *xlnB* and *xlnD* [58].

Regarding the expression regulation of the transporter and the α-L-rhamnosidase genes mediated by RhaR, we also analyzed the microarray expression data of *A*. *niger* WT and a Δ*rhaR* strain grown on L-rhamnose (accession number GSE51023) recently deposited by Gruben *et al* at GEO [18], observing that in this independent study using a different setup *rhtA* (An12g05710) and *rhaB* (An12g05700) were not induced in a RhaR knockout strain. Therefore, both microarray (Gruben *et al*) and RT-qPCR (this study) data analysis of two independent studies indicated that RhaR is responsible for *rhtA* and *rhaB* transcriptional activation. However, although the effect produced by RhaR in the regulation of these genes is very clear, as can be observed in Fig 7 their induction was not fully abolished in the Δ*rhaR* strain. This might indicate the influence of another transcriptional regulator that responds to the presence of L-rhamnose. Previously, a second transcriptional factor (FST14) was suggested to be involved in the regulation of the *P*. *stipitis* pectinolytic network [16], and this could also be the case in *A*. *niger*, as recently suggested by Gruben *et al* [18].

In the presence of L-rhamnose plus D-glucose, *rhtA* and *rhaB* were heavily repressed in the wild type strain, and only partly derepressed in the Δ*creA* mutant. While this result suggests a role of CreA in controlling the expression of *rhtA* and *rhaB*, as reported before for other sugar utilisation systems [11,46,59,60], a different glucose-repression mechanism seems to have a major role in the transcriptional regulation of these genes. The existence of CreA-independent glucose repression mechanisms controlling the regulation of α-L-rhamnosidase genes [13], and the phenylacetic acid uptake system [61], has been previously reported in *A*. *nidulans*.

The isolation of *rhtA* and *rhaB* deletion strains allowed us to investigate the relevance of both proteins in *A*. *niger*. On one hand, the absence of RhtA produced a growth and sporulation defect in the Δ*rhtA* mutants on L-rhamnose containing media, underpinning the relevant biological role of RhtA for transport of L-rhamnose. Similar phenotype analyses performed in filamentous fungi, where different sugar transporter mutant strains were studied, produced disparate results. In some cases, an altered growth phenotype could not be detected in plate assays containing the transporter’s specific substrate [30,55,62,63], which is probably due to overlapping substrate specificities. In this regard, the most notorious case corresponds to *S*. *cerevisiae*, where many genes had to be disrupted before its ability to transport D-glucose was abolished [29]. However, as we observed in the present study, the absence of certain transporters in filamentous fungi has also been shown to be accompanied with clear growth defects [64–66]. On the other hand, the absence of *rhaB* did not affect negatively the mutant growth in the presence of RG1. This result is not surprising, bearing in mind that α-L-rhamnosidase genes appear to be quite redundant in the *A*. *niger* genome.

RhtA is, according to our knowledge, the first functionally validated eukaryotic transporter containing a FucP domain structure. It is also the first eukaryotic L-rhamnose transporter functionally validated to date, therefore this study provides major insights about the utilisation of this monosaccharide by fungi. The identification of RhtA will also have an impact in the design of new microbial strains using L-rhamnose-rich biomass as feedstocks, like pectic polysaccharides from plants [67], or ulvan from green seaweeds [23,68], for the production of fuels and chemicals.

## Materials and Methods

### Strains and growth conditions

The *Aspergillus niger* strains used in this study were N400 (NRRL3, ATCC9029, CBS120.49), N402 (*cspA1*) [69], NW283 (*fwnA1; cspA1; lysA7 pyrA6; creAd4*) [59], MA169.4 (*cspA1*, kusA::DR-*amdS*-DR, *pyrG*^−^) [70], and its derivatives JS14, (Δ*rhaR*), JS16 (Δ*rhtA*) and JS19 (Δ*rhaB*) constructed in this study. *A*. *niger* spores were generated on complete medium (CM) plates. Mycelial biomass for transfer experiments was produced in 18 hour pre-cultures after the inoculation of 10^6^ spores ml^-1^ in culture medium containing: 6 g L^−1^ (w/v) NaNO_3_, 1.5 g L^−1^ (w/v) KH_2_PO_4_, 0.5 g L^−1^ (w/v) KCl, 0.5 g L^−1^ (w/v) MgSO_4_H_2_O and Vishniac salts [71,72], with 5 g L^−1^ (w/v) yeast extract, D-sorbitol 100 mM, and the appropriate supplements to complement auxotrophic mutations (initial pH 6.0).

For the plasma membrane proteomics analysis, equal amounts of water-rinsed mycelium of the wild type strain *A*. *niger* N400 were transferred to 1-liter benchtop fermenters (Sartorius) with 750 mL of minimal medium (MM) containing: 6 g L^−1^ (w/v) NaNO_3_, 1.5 g L^−1^ (w/v) KH_2_PO_4_, 0.5 g L^−1^ (w/v) KCl, 0.5 g L^−1^ (w/v) MgSO_4_H_2_O, Vishniac salts, and one of the following carbon sources: L-arabinose 5 mM, D-mannose 5 mM, D-sorbitol 100 mM plus D-xylose 0.1 mM, and L-rhamnose 5 mM. Two biological replicates per condition were studied. Fermenters were kept at 30°C, stirred at 1000 rpm and aerated with filtered air (0.6 L min^−1^), keeping oxygen levels over 60%. The initial pH, set at 4.0, was allowed to drop until pH 3.5 and kept constant afterwards by sodium hydroxide addition. These culture conditions were also used in the study of *rhtA* transcriptional response to D-sorbitol 100 mM, L-rhamnose 5 mM, and D-fructose 5 mM; and also in the time course fermentations in cultures containing D-sorbitol 100 mM, L-rhamnose 1 mM, or L-rhamnose 5 mM.

For the analysis of the regulatory mechanisms controlling *rhtA* and *rhaB*, the *A*. *niger* mycelial biomass of the strains N402, NW283 (Δ*creA*) and JS14 (Δ*rhaR*) was produced in 18 hour pre-cultures (2 Erlenmeyer flasks per strain) after the inoculation of 10^6^ spores ml^-1^ in culture medium containing: 6 g L^−1^ (w/v) NaNO_3_, 1.5 g L^−1^ (w/v) KH_2_PO_4_, 0.5 g L^−1^ (w/v) KCl, 0.5 g L^−1^ (w/v) MgSO_4_H_2_O, Vishniac salts, 1 g L^−1^ (w/v) yeast extract, 100 mM D-sorbitol, and the appropriate supplements to complement auxotrophic mutations (initial pH 6.0). Equal amounts of mycelium from each strain were then transferred to 100 mL Erlenmeyer flasks with the same medium composition, containing 5 mM L-rhamnose or 5 mM L-rhamnose plus 50 mM D-glucose as carbon sources. Cultures were performed in an orbital shaker for two hours at 30°C and 225 rpm.

The phenotype analysis of the different *A*. *niger* mutant strains obtained in this study was done in agar plates, containing MM plus the appropriate supplements and carbon sources.

The *S*. *cerevisiae* strain EBY.VW4000 (CEN.PK2-1C *hxt13*Δ::*loxP*; *hxt15*::Δ*loxP*; *hxt16*Δ::*loxP*; *hxt14*Δ::*loxP*; *hxt12*Δ::*loxP*; *hxt9*Δ::*loxP*; *hxt11*Δ::*loxP*; *hxt10*Δ::*loxP*; *hxt8*Δ::*lox*P; *hxt514*Δ::*loxP*; *hxt2*Δ::*loxP*; *hxt367*Δ::*loxP*; *gal2*Δ; *stl1*Δ::*loxP*; *agt1*::*loxP*; *ydl247w*Δ::*loxP*; *yjr160c*Δ::*loxP*) [29], that was used in this study for the functional validation of the *rhtA* gene, was grown at 30°C and maintained in solid complete medium containing 10 g L^−1^ (w/v) of yeast extract, 20 g L^−1^ (w/v) of peptone and 20 g L^−1^ (w/v) of maltose. The EBY.VW4000 derived strains obtained in the present study where grown in liquid minimal medium (MM) containing 6.7 g L^−1^ (w/v) of yeast nitrogen base with ammonium sulphate (w/o amino acids) (Difco), supplemented with leucine (30 mg L^−1^; w/v), tryptophan (20 mg L^−1^; w/v) and histidine (20 mg L^−1^; w/v), and using 20 g L^−1^ (w/v) of maltose as carbon source. The *S*. *cerevisiae* wild type strain CEN.PK2-1C (*MATα; his3Δ1*; *leu2-3*_*112*; *ura3-52*; *trp1-289*; *MAL2-8*^*c*^; *SUC2*) strain was used as a control strain.

For the yeast spot assays, the different *S*. *cerevisiae* strains were grown overnight as mentioned above and harvested in exponential phase by centrifugation. Cells were then diluted to the following optical densities at 600 nm (OD600): 1, 0.1, 0.01 and 0.001, and subsequently 5 μl droplets were spotted on 1.5% agar plates containing MM plus the appropriate supplements and different carbon sources.

For radiolabelled sugars uptake experiments *S*. *cerevisiae* strains were cultured in baffled flasks with MM supplemented with methionine and arginine for enhanced growth, and histidine, leucine and tryptophan to complement auxotrophic mutations. Incubations were done in an orbital shaker at 30°C and 225 rpm.

### *A*. *niger* membrane associate proteome purification, quality control analysis, sample preparation for LC-MS/MS and proteomics data analysis

The *A*. *niger* membrane proteome preparation and purification was performed as described [36]. *A*. *niger* mycelium samples (2–3 g, press-dried), washed and resuspended in 20 mM HEPES buffer (pH 7.6) containing 150 mM NaCl and protease inhibitor cocktail for yeast & fungi (Sigma-Aldrich), were mechanically disrupted using a French press (8000 psi). Three differential centrifugation steps, at low (500 g), medium (5,000 g) and high speed (~85,000 g), were performed to pellet light organelles (P3). P3 pellets were resuspended using a Dounce homogenizer in 1 mL of 20 mM HEPES buffer (pH 7.6), containing 250 mM sucrose. The P3 suspensions were subsequently overlaid in a discontinuous sucrose density gradient, prepared by layering successive solutions, with decreasing sucrose concentrations (6 x 1 mL; 1.20 M to 0.70 M), upon one another. Sucrose density gradients were centrifuged (~100,000 g– 60 min) to isolate different membrane-associated fractions from P3 pellet. Five fractions were obtained (P3A, P3B, P3C, P3D and P3E). The plasma membrane (PM) marker vanadate-sensitive H+ ATPase and the mitochondrial membrane cytochrome c oxidase activities were then measured in the initial cell free extract, the P3 pellet and the P3A to P3E fractions derived from it. Compared to the cell free extract, the P3 pellet was 2.4 to 3.2 times enriched in plasma membranes. No further enhanced PM enrichment was found in the P3A to P3E fractions, therefore the P3 pellets were considered to be more optimal for the analysis of plasmalemma proteins, that were further processed and subjected to shotgun proteomics analysis. Cytochrome c oxidase activity measurements were performed using the “Cytochrome c Oxidase Assay Kit” from Sigma-Aldrich (CYTOCOX1), following the user manual. The vanadate-sensitive H+ ATPase enzyme assay was performed as described previously [73].

The protocol used in order to prepare membrane proteins for LC-MS/MS analysis has been described in detail previously [36]. LC-MS/MS analyses were done at Radboud Proteomics Centre as described previously [74]. The obtained raw mass spectrometry proteomics data was deposited to the ProteomeXchange Consortium via the PRIDE partner repository with the dataset identifier PXD004909. The analysis of the LC-MS/MS spectra obtained from the proteomics experiment, were identified and quantified using the MaxQuant software [75], as described [36].

### Construction of *S*. *cerevisiae* EBY.VW4000 strain expressing *A*. *niger rhtA* gene

The chemically synthesized *rhtA* cDNA coding sequence (*A*. *niger* ATCC 1015 JGI prot. ID: 1119135) was PCR amplified from plasmid using primers HE_*rhtA*_FW and HE_*rhtA*_RV (S2 Table), using Phusion polymerase (ThermoFisher Scientific) following the manufacturers protocol. The fragment was then digested with *Spe*I and *Xho*I and cloned into the *S*. *cerevisiae* expression vector p426HXT7-6His, linearized with the same restriction enzymes, under the control of the constitutive promoter HXT7p and the terminator CYC1t. Transformation of *S*. *cerevisiae* EBY.WV4000 was performed as described previously [76].

### Construction of JS14 (Δ*rhaR*), JS16 (Δ*rhtA*) and JS19 (Δ*rhaB*)

Using the split-marker approach, the previously identified L-rhamnose regulator gene *rhaR* (JGI ATCC 1015 Prot ID 1116273) [18], the rhamnose transporter gene *rhtA* (JGI ATCC 1015 Prot ID 1119135), or the putative rhamnosidase gene *rhaB* (JGI ATCC 1015 Prot ID 131668) were deleted from the genome of the MA169.4 strain (isogenic of N402), which is defective in the Non-Homologous End-Joining (NHEJ) pathway through a transiently silenced *kusA* gene [70,77]. A schematic representation of the four experimental steps required can be found in S3 Fig, PCR results that confirm the correct deletion of the genes from the genome can be found in S4 Fig and primers used are listed in S2 Table.

As an example we describe the construction of JS14 (Δ*rhaR*) knockout strains, the other strains were constructed in the same manner with the corresponding primers. First, homologous regions were amplified from *A*. *niger* N402 genomic DNA using primers KO_*rhaR*5’_FW with KO_*rhaR*5’_RV and KO_*rhaR*3’_FW with KO_*rhaR*3’_RV, and the marker gene was amplified from pAO4-13 using primers KO_*pyrG*_FW and KO_*pyrG*_RV. Second, these three fragments were used as template to create marker-flank fusion fragments using primers KO_*rhaR*5’_FW with KO_*pyrG*2_RV and KO_*pyrG*2_FW with KO_*rhaR*3’_RV. Third, the resulting fragments were used to transform MA169.4 as previously described [78]. Single *A*. *niger* transformant colonies were purified and the transiently silenced *kusA* gene was restored on MM plates containing fluoroacetic acid (FAA). Finally, correct marker localization in the strain JS14 (*ΔrhaR*) was checked by PCR using genomic DNA as template, and the primer pairs CH_locus*rhaR*_FW with KO_*pyrG*2_RV and KO_*pyrG*2_FW with CH_locus*rhaR*_RV. Deletion of the *rhaR* gene was confirmed by PCR using the primers CH_*rhaR*_FW and CH_*rhaR*_RV.

### RNA extraction and transcriptional analysis of *rhtA* and *rhaB* genes

Mycelium samples were disrupted with glass beads in a Fastprep-24 instrument, and RNA was isolated using a Maxwell 16 instrument using the Maxwell 16 LEV simplyRNA kit (Promega). Reverse transcription and qPCR analysis were performed following the protocols and instruments described in Mach-Aigner et al., 2012 [79]. In short, after treatment with DNase I, cDNA was synthesized from 0.45 μg RNA using the RevertAid H Minus First Strand cDNA synthesis kit (Thermo Fisher). All reactions were performed according to the manufacturer’s instructions. All quantitative PCRs (qPCRs) were performed in triplicate in a Rotor-Gene 3000 cycler (Qiagen). The amplification mixture (final volume, 15 μl) contained 7.5 μl of 2x ABsolute QPCR SYBR Green mix, 100 nM forward and reverse primers and 2.5 μl cDNA (diluted 1:100). The primers used for qPCR analysis were designed using the software QuantPrime [80], and are listed in S2 Table. Each run included a no-template control and a no-amplification control (0.015% SDS added to the reaction mixture). The cycling conditions comprised a 15 min initial polymerase activation at 95°C, followed by 40 cycles of 95°C for 15 s, 59°C for 15 s, and 72°C for 15 s. The previously described histone-like gene *hist* transcript (*A*. *niger* ATCC 1015 gene ID 207921) and the Golgi Transporter gene (*A*. *niger* CBS 513.88 An02g04120) were used as reference for normalization of the expression data [58,79]. Dissociation (or melting) curve analysis was performed on each qPCR reaction to confirm that the primer pairs used produced a single amplification product. Results are given as relative transcript ratios in logarithmic scale (lg(10)). The values provided in the figures correspond to two biological replicates per strain and culture condition.

### Sugars analysis

Sugars present in the *A*. *niger* culture supernatants were measured through high-pressure liquid chromatography (HPLC) analysis. A Thermo Accela equipped with a Shodex KC-811 column, coupled to a refractive index detector (Spectrasystem RI-150, sample frequency 5.00032 Hz) and a UV-VIS detector (Spectrasystem UV1000, λ: 210 nm), was used. Separations were performed by isocratic elution with 0.01 N H_2_SO_4_, at a flow rate of 0.8 mL min^-1^. Crotonate (6 mM) was used as an internal standard.

### Radiolabeled sugar uptake determinations

Sugar uptake assays were performed as described previously, with minor adjustments [81]. Liquid cultures using MM, supplemented with methionine (20 mg L^−1^; w/v), arginine (20 mg L^−1^; w/v), leucine (30 mg L^−1^; w/v), tryptophan (20 mg L^−1^; w/v) and histidine (20 mg L^−1^; w/v), with 1% (w/v) maltose as carbon source, were inoculated with strains EBY.VW4000_XltB (control), and EBY.VW4000_RhtA, and incubated for 5 days. Cells were then harvested by centrifugation (4000 g, 10 min), washed with 50 mL ice-cold ultrapure water, and washed again with ice-cold PBS (pH 6.5). Cells were then resuspended in PBS (pH 6.5), divided in 40 μL aliquots, and kept on ice.

Aliquots were incubated for 5 min at 30°C before the uptake assay was started. To start the assay, 10 μl of a 100 μM L-[^3^H(G)]-rhamnose, D-[^14^C(U)]-fructose or D-[1-^14^C]-xylose solution (Campro Scientific, Veenendaal) was added. After exactly 20 seconds the reaction was quenched by the addition of 1 mL of ice-cold wash buffer (PBS, pH 6.5, with 500 mM of the corresponding non-labeled substrate solution), followed by a vacuum filtration step (0.45 μm HV filters, 1225 sampling manifold, Millipore), and two subsequent washing steps with 5 mL of ice-cold washing buffer. After drying for 5 min in the vacuum manifold, the filters were transferred to scintillation vials with 5 mL scintillation liquid (Ultima Gold, Perkin Elmer), and activity was counted (Packard Tricarb 1600TR). All reactions were performed in triplicate. Negative control reactions, where quenching was done before substrate addition and without incubation, were performed for each reaction.

### Bioinformatic analysis

DNA and protein sequences were obtained from the JGI *A*. *niger* ATCC 1015 genome database [37]. Retrieved sequences were subsequently used in additional searches using the BLAST tools at the NCBI database. Protein transmembrane-aware multiple alignments were done using a PRALINE, incorporating TMHMM2.0 transmembrane helix prediction tool [82,83]. SignalP4.1 was used to detect the signal peptide for secretion in the RhaB protein [84].

## Supporting Information

S1 Dataset Relative abundances of proteins from the *A*. *niger* membrane-associated proteome detected in four different growth conditions. (XLSX)

S1 Fig Sugar concentration analysis (HPLC) of culture supernatants at two different sampling points (t = 0h and 2 h) during *A*. *niger* N400 fermentations for plasma membrane proteomic analysis. (PDF)

S2 Fig Time course transcriptional analysis of *rhtA*. Relative transcription levels, measured by RT-qPCR, of *rhtA* during *A*. *niger* N400 fermentations in minimal medium with an initial concentration of L-rhamnose 1 mM (black bars), L-rhamnose 5 mM (grey bars), or D-sorbitol 100 mM (white bars). Transcript levels of *rhaA* always refer to the reference sample (D-sorbitol 100 mM; t = 1h). The values provided in the figures correspond to two biological replicates per culture condition. Error bars are means of three technical replicates.
(PDF)

S3 Fig Construction of deletion strains. Schematic representation of experimental steps taken for deletion of *rhaR*, *rhtA* and *rhaB* from the *A*. *niger* genome.
(PDF)

S4 Fig Confirmation by PCR of the correct integration of the marker gene and deletion of *rhaR*, *rhtA* and *rhaB* genes, as described in the Materials and Methods section, and S3 Fig. Small codes above the gels indicate: L = ladder; T1 = Transformant 1; T2 = Transformant 2; WT = N402; (-) = blank/no template control
(PDF)

S1 Table *A*. *niger* RhtA Bi-directional Best BLAST Hits with fungi belonging to different fungal orders. (DOCX)

S2 Table Primers used in this study. (DOCX)
