# Supplementary figures and images for: Identification of a Novel L-rhamnose Uptake Transporter in the Filamentous Fungus Aspergillus niger
Source: PLoS Genet. 2016 Dec 16;12(12):e1006468. doi: 10.1371/journal.pgen.1006468 (PMC5161314; doi:10.1371/journal.pgen.1006468)

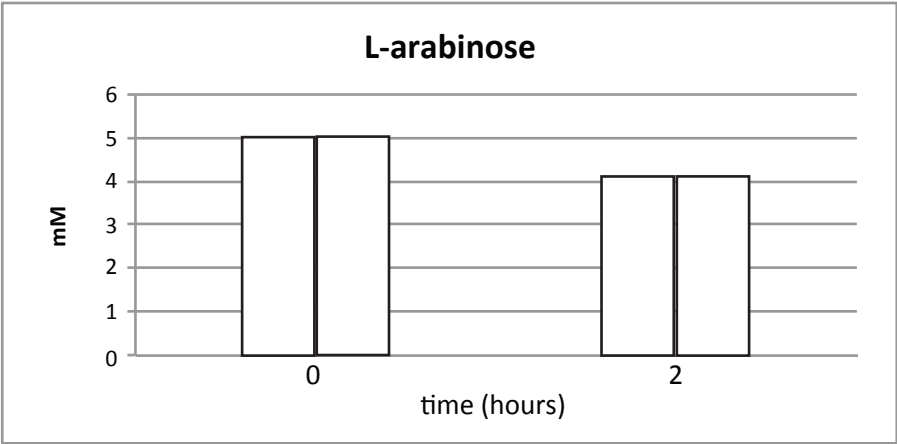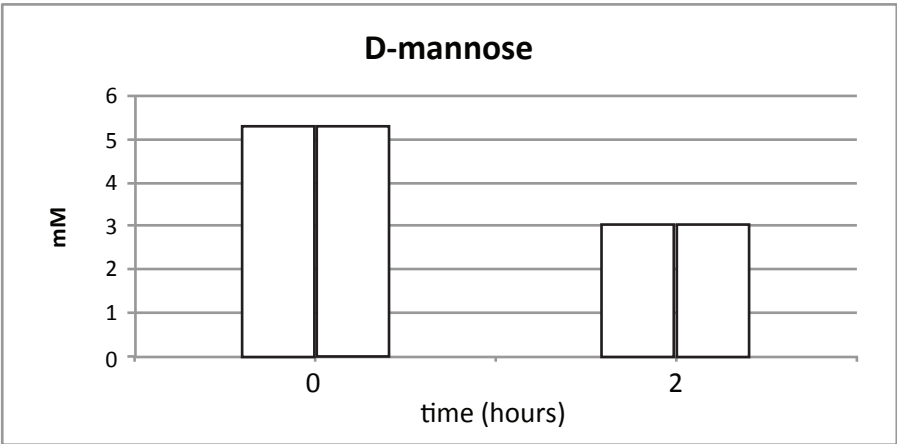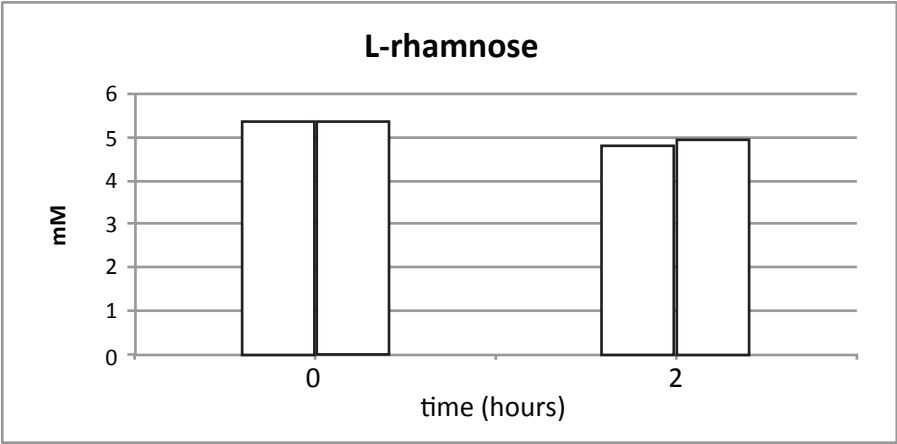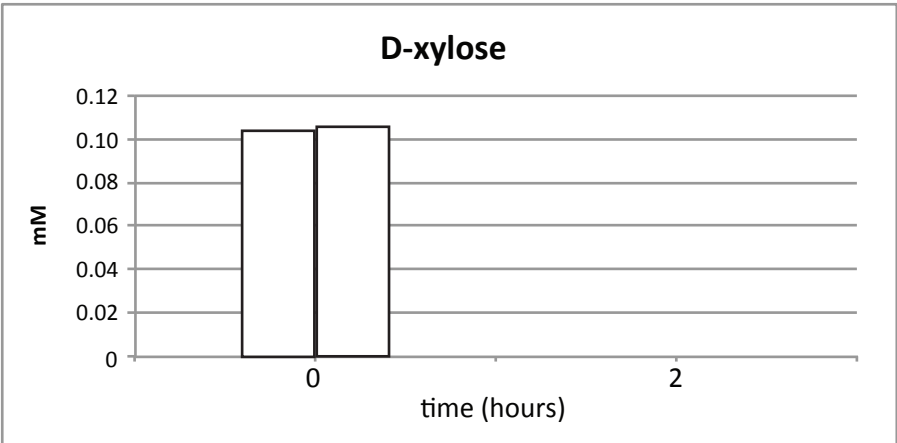

Supplement: S1 Fig — (PDF) [file pgen.1006468.s002.pdf]

# *rhtA*

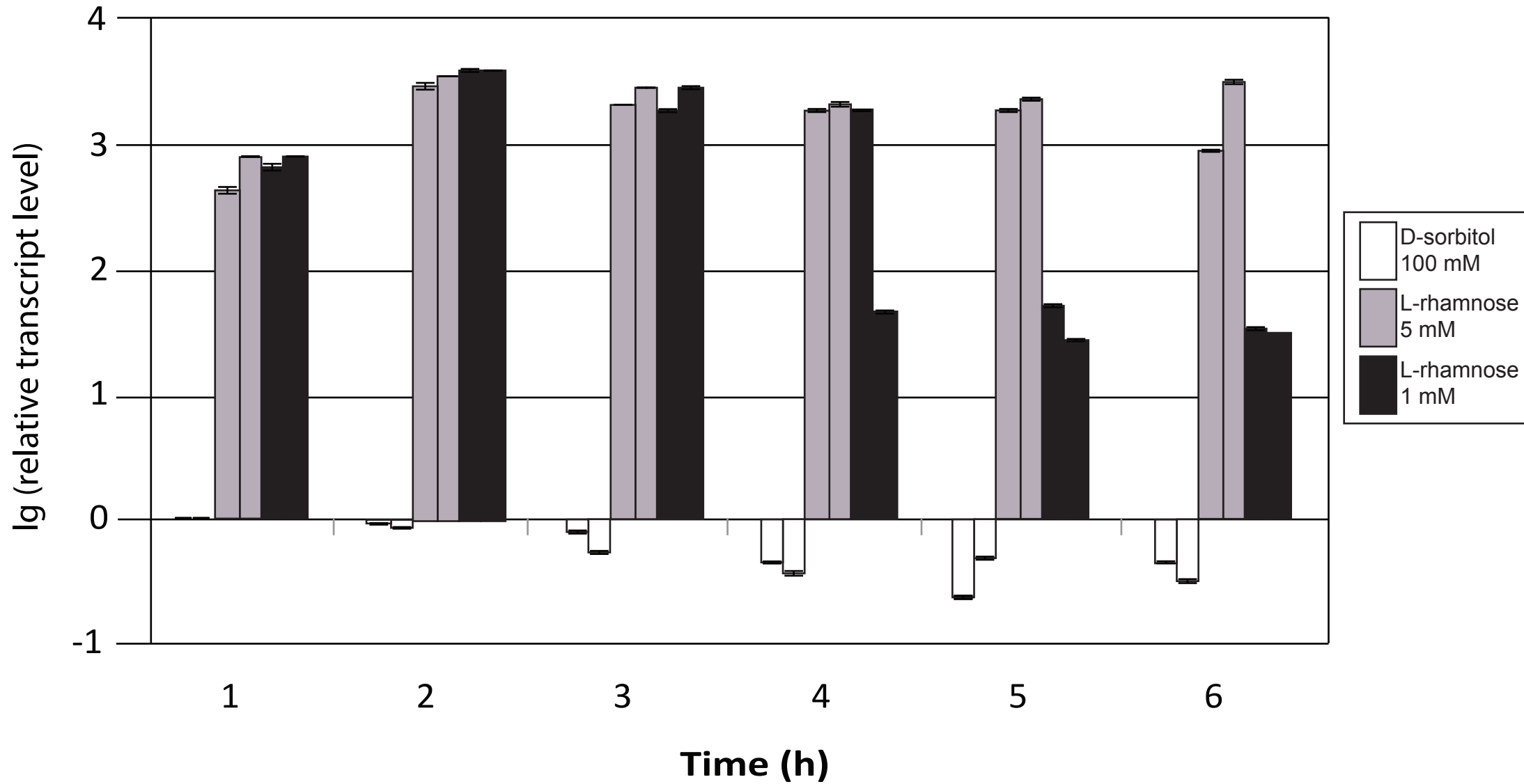

Supplement: S2 Fig — Relative transcription levels, measured by RT-qPCR, of rhtA during A. niger N400 fermentations in minimal medium with an initial concentration of L-rhamnose 1 mM (black bars), L-rhamnose 5 mM (grey bars), or D-sorbitol 100 mM (white bars). Transcript levels of rhaA always refer to the reference sample (D-sorbitol 100 mM; t = 1h). The values provided in the figures correspond to two biological replicates per culture condition. Error bars are means of three technical replicates. (PDF) [file pgen.1006468.s003.pdf]

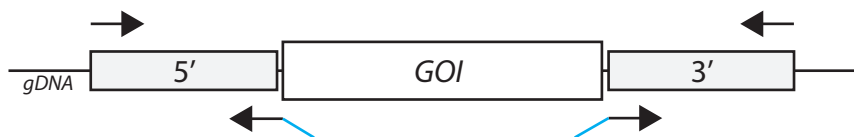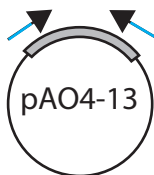

1

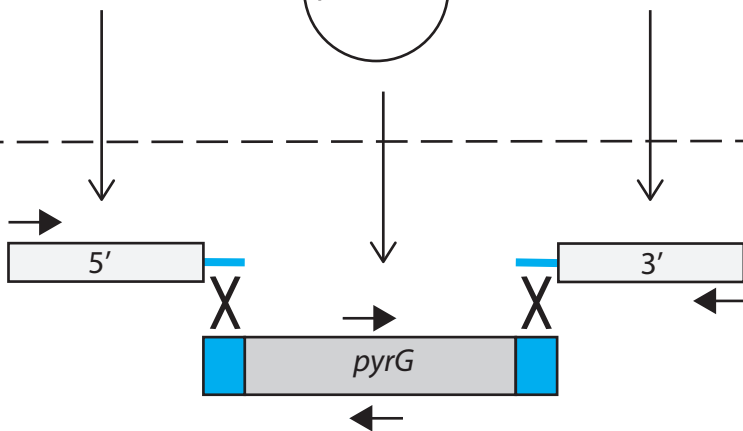

2

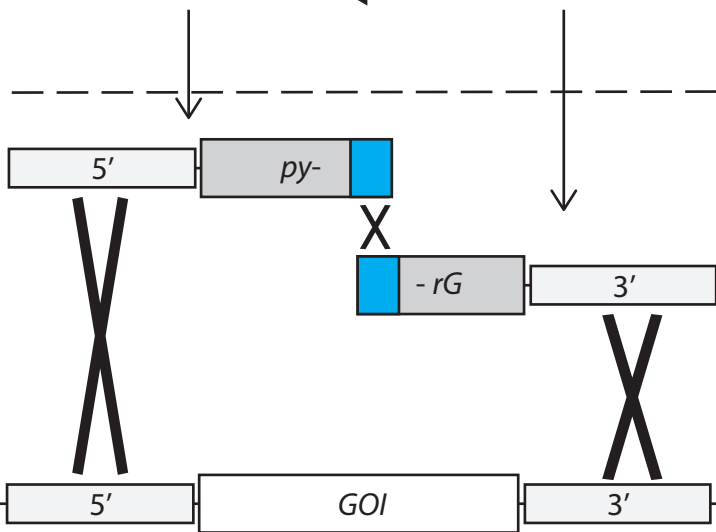

3

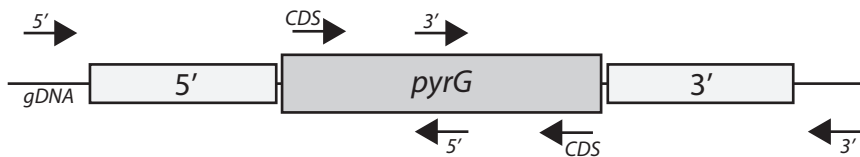

4

Supplement: S3 Fig — Schematic representation of experimental steps taken for deletion of rhaR, rhtA and rhaB from the A. niger genome. (PDF) [file pgen.1006468.s004.pdf]

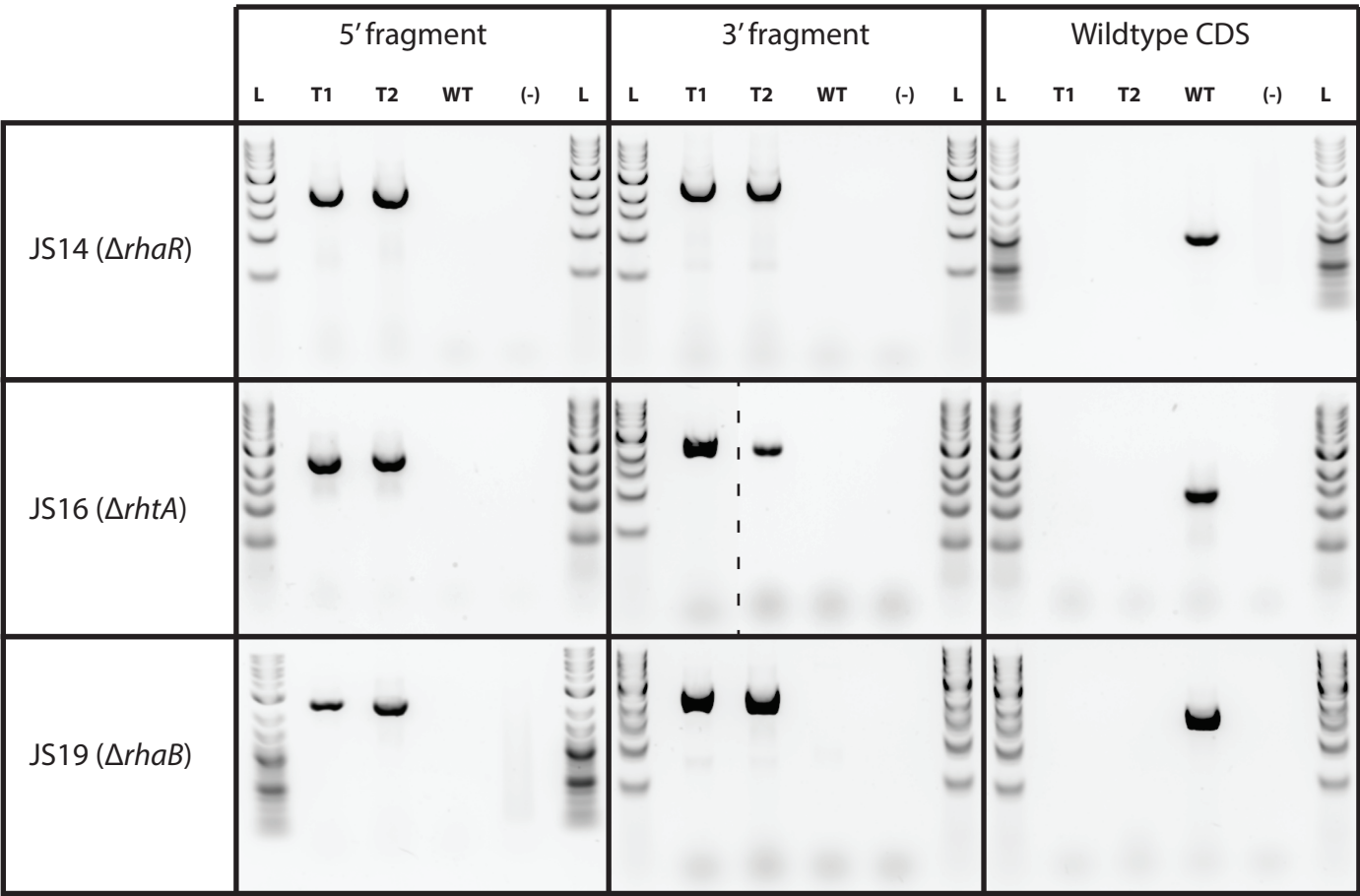

Supplement: S4 Fig — Small codes above the gels indicate: L = ladder; T1 = Transformant 1; T2 = Transformant 2; WT = N402; (-) = blank/no template control (PDF) [file pgen.1006468.s005.pdf]
