# Supplementary material for: Identification of a Novel L-rhamnose Uptake Transporter in the Filamentous Fungus Aspergillus niger
Source: PLoS Genet. 2016 Dec 16;12(12):e1006468. doi: 10.1371/journal.pgen.1006468 (PMC5161314; doi:10.1371/journal.pgen.1006468)
Supplement: S1 Table — (DOCX) [file pgen.1006468.s006.docx]

| **Order** | **Protein GI** | **Species** |
| --- | --- | --- |
| Botryosphaeriales | 821063869 | *Diplodia seriata* |
|  | 615430705 | *Neofusicoccum parvum* |
|  | 407928841 | *Macrophomina phaseolina* |
|  | 615409976 | *Neofusicoccum parvum* |
|  | 821064401 | *Diplodia seriata* |
| Capnodiales | - | *-* |
| Diaporthales | 972129535 | *Valsa mali* |
|  | 821076379 | *Diaporthe ampelina* |
| Dothidealis | 662507865 | *Aureobasidium elanogenum* |
|  | 918828118 | *Aureobasidium namibiae* |
|  | 662528692 | *Aureobasidium pullulans* |
|  | 915703626 | *Aureobasidium subglaciale* |
|  | 662503751 | *Aureobasidium elanogenum* |
|  | 915692983 | *Aureobasidium subglaciale* |
|  | 918818940 | *Aureobasidium namibiae* |
| Erysiphales | 730185595 | *Erysiphe necator* |
| Eurotiales | 350636973 | *Aspergillus niger* |
|  | 358369948 | *Aspergillus kawachii* |
|  | 1002322763 | *Aspergillus luchuensis* |
|  | 915139352 | *Rasamsonia emersonii* |
|  | 816192534 | *Talaromyces islandicus* |
|  | 700461016 | *Penicillium expansum* |
|  | 700494227 | *Penicillium italicum* |
|  | 972237340 | *Aspergillus calidoustus* |
|  | 816346193 | *Aspergillus ochraceoroseus* |
|  | 816341999 | *Aspergillus rambellii* |
|  | 242780494 | *Talaromyces stipitatus* |
| Glomerellales | 530473699 | *Colletotrichum gloeosporioides* |
|  | 380492229 | *Colletotrichum higginsianum* |
|  | 477528947 | *Colletotrichum orbiculare* |
|  | 640921730 | *Colletotrichum sublineola* |
|  | 615444814 | *Colletotrichum fioriniae* |
|  | 996614254 | *Colletotrichum nymphaeae* |
|  | 996628185 | *Colletotrichum salicis* |
| Helotiales | 636749063 | *Glarea lozoyensis* |
|  | 156039225 | *Sclerotinia sclerotiorum* |
|  | 563289507 | *Sclerotinia borealis* |
|  | 972531614 | *Phialocephala scopiformis* |
|  | 472236527 | *Botrytis cinerea* |
|  | 597575965 | *Marssonina brunnea* |
| Hypocrealis | 667528564 | *Stachybotrys chartarum* |
|  | 667717948 | *Stachybotrys chlorohalonata* |
| Magnaporthales | 835892707 | *Magnaporthiopsis poae* |
|  | 389641001 | *Magnaporthe oryzae* |
|  | 685405941 | *Gaeumannomyces graminis* |
| Microascales | 666867100 | *Scedosporium apiospermum* |
|  | 802102361 | *Thielaviopsis punctulata* |
| Onygenales | - | *-* |
| Ophiostomatales | 930147586 | *Grosmannia clavigera* |
|  | 512189469 | *Ophiostoma piceae* |
| Orbiliales | 748503496 | *Dactylellina haptotyla* |
|  | 582956063 | *Drechslerella stenobrocha* |
|  | 748501463 | *Arthrobotrys oligospora* |
| Pezizales | - | *-* |
| Pleosporales | 909990832 | *Stemphylium lycopersici* |
|  | 189210704 | *Pyrenophora tritici-repentis* |
|  | 330925441 | *Pyrenophora teres* |
|  | 928519804 | *Bipolaris maydis* |
|  | 953438543 | *Bipolaris victoriae* |
|  | 628185892 | *Bipolaris zeicola* |
|  | 627922415 | *Bipolaris oryzae* |
|  | 628071521 | *Bipolaris sorokiniana* |
|  | 396473670 | *Leptosphaeria maculans* |
|  | 636595563 | *Setosphaeria turcica* |
|  | 169594578 | *Parastagonospora nodorum* |
| Saccharomycetales | 667528564 | *Stachybotrys chartarum* |
|  | 667717948 | *Stachybotrys chlorohalonata* |
| Sordariales | 1003747199 | *Madurella mycetomatis* |
|  | 171679429 | *Podospora anserina* |
| Xylariales | 630015337 | *Pestalotiopsis fici* |
