# Supplementary material for: Identification of a Novel L-rhamnose Uptake Transporter in the Filamentous Fungus Aspergillus niger
Source: PLoS Genet. 2016 Dec 16;12(12):e1006468. doi: 10.1371/journal.pgen.1006468 (PMC5161314; doi:10.1371/journal.pgen.1006468)
Supplement: S2 Table — (DOCX) [file pgen.1006468.s007.docx]

| **Name** | **Sequence** |
| --- | --- |
| HE*_rhtA*_FW | GAGAACTAGTATGTGGCGAAGACGCATCAC |
| HE*_rhtA*_RV | GAGACTCGAGTTACGCAGCATTGATATTCTCCAC |
| KO_*rhaR*5’_FW | CATGGGGGTGGAGATGTTAC |
| KO_*rhaR*5’_RV | CAATTCCAGCAGCGGCTTCGGGGTTTATCGGGAGATAG |
| KO_*rhaR*3’_FW | ACACGGCACAATTATCCATCGTGTTCACCATGCCGTACACT |
| KO_*rhaR*3’_RV | CTTCACGATCATGGCAGCTA |
| CH_locus*rhaR*_FW | AGCCCAGGACGTTAATACCC |
| CH_locus*rhaR*_RV | TTGTCCGAGTTGAGCCTTCT |
| CH_*rhaR*_FW | CAACACCGTCGACACACAAC |
| CH_*rhaR*_RV | GCAATTCCACCTCCACCTCA |
| KO_*rhtA*5’_FW | ATGAAACGAGCGTGCAGTTG |
| KO_*rhtA*5’_RV | CAATTCCAGCAGCGGCTTGTTGGGCTATCGCGGAAG |
| KO_*rhtA*3’_FW | ACACGGCACAATTATCCATCGGGGATGCAGTCCTGTGTCAG |
| KO_*rhtA*3’_RV | GTCGAACCGCCTTCATCATT |
| CH_locus*rhtA*_FW | GCGCCGACGTATGTTTTCTC |
| CH_locus*rhtA*_RV | GGACTGATTGGTACCGCTCCT |
| CH_*rhtA*_FW | TGCCCCCTAACAGTCTCTGG |
| CH_*rhtA*_RV | ATCATGGCCATCCACAACCT |
| KO_*rhaB*5’_FW | CGACATAACCGGAGCTTTGT |
| KO_*rhaB*5’_RV | CAATTCCAGCAGCGGCTTGGCACCCGTAGCTGTCAAGG |
| KO_*rhaB*3’_FW | ACACGGCACAATTATCCATCGGCCTATCTCGAGACAATTTC |
| KO_*rhaB*3’_RV | TGGGAGTTGAGGGTATCGAG |
| CH_locus*rhaB*_FW | AGCATGCTGTCCCTTCAGTT |
| CH_locus*rhaB*_RV | AATCGGCGGAGAATCCAC |
| CH_*rhaB*_FW | CTTTCCAACACCTTCCGTGT |
| CH_*rhaB*_RV | GAAAGGCGCAAGATTAGTCG |
| KO_*pyrG*_FW | AAGCCGCTGCTGGAATTG |
| KO_*pyrG*_RV | CGATGGATAATTGTGCCGTGT |
| KO_*pyrG*2_FW | ATTGACCTACAGCGCACGC |
| KO_*pyrG*2_RV | CCGGTAGCCAAAGATCCCTT |
| QP_*hist*_FW | ATCTTGCGTGACAACATCCA |
| QP_*hist*_RV | CACCCTCAAGGAAGGTCTTG |
| QP_*GoTra*_FW | TCCTGGGTCATGTCACCATTGC |
| QP_*GoTra*_RV | TCTTGGGCAGAAGGTTCTGGTG |
| QP_*rhtA*_FW | TGGTCCAACCAGTGTCGCTATG |
| QP_*rhtA*_RV | TTGTGGCGAAGCAGGCACTTTC |
| QP_*rhaB*_FW | ACTGGCACGACCTGGGAATATC |
| QP_*rhaB*_RV | GTGCGCATGCGACGTATAGAAG |

HE = Heterologous expression in yeast

KO = Knockout

CH = Check

QP = RT-qPCR
